# Rotavirus NSP4: Cell type-dependent transport kinetics to the exofacial plasma membrane and release from intact infected cells

**DOI:** 10.1186/1743-422X-8-278

**Published:** 2011-06-06

**Authors:** Thomas F Gibbons, Stephen M Storey, Cecelia V Williams, Avery McIntosh, DeAnne M Mitchel, Rebecca D Parr, Megan E Schroeder, Friedhelm Schroeder, Judith M Ball

**Affiliations:** 1 Department of Pathobiology Texas A&M University, TVMC, College Station, TX 77843-4467, USA; 2 Departments of Physiology and Pharmacology, Texas A&M University, TVMC, College Station, TX 77843-4466, USA; 3 Lackland Air Force base, San Antonio, TX, USA; 4 Physiology and Pharmacology, Texas A&M University, College Station, TX 77843-4466, USA; 5 Sandia National Laboratories, Albuquerque, New Mexico, USA; 6 Arkansas State University, Jonesboro, AR 72467, USA; 7 Texas State Veterinary Diagnostic Laboratory, College Station, TX 77843, USA

## Abstract

**Background:**

Rotavirus NSP4 localizes to multiple intracellular sites and is multifunctional, contributing to RV morphogenesis, replication and pathogenesis. One function of NSP4 is the induction of early secretory diarrhea by binding surface receptors to initiate signaling events. The aims of this study were to determine the transport kinetics of NSP4 to the exofacial plasma membrane (PM), the subsequent release from intact infected cells, and rebinding to naïve and/or neighboring cells in two cell types.

**Methods:**

Transport kinetics was evaluated using surface-specific biotinylation/streptavidin pull-downs and exofacial exposure of NSP4 was confirmed by antibody binding to intact cells, and fluorescent resonant energy transfer. Transfected cells similarly were monitored to discern NSP4 movement in the absence of infection or other viral proteins. Endoglycosidase H digestions, preparation of CY3- or CY5- labeled F(ab)_2 _fragments, confocal imaging, and determination of preferential polarized transport employed standard laboratory techniques. Mock-infected, mock-biotinylated and non-specific antibodies served as controls.

**Results:**

Only full-length (FL), endoglycosidase-sensitive NSP4 was detected on the exofacial surface of two cell types, whereas the corresponding cell lysates showed multiple glycosylated forms. The C-terminus of FL NSP4 was detected on exofacial-membrane surfaces at different times in different cell types prior to its release into culture media. Transport to the PM was rapid and distinct yet FL NSP4 was secreted from both cell types at a time similar to the release of virus. NSP4-containing, clarified media from both cells bound surface molecules of naïve cells, and imaging showed secreted NSP4 from one or more infected cells bound neighboring cell membranes in culture. Preferential sorting to apical or basolateral membranes also was distinct in different polarized cells.

**Conclusions:**

The intracellular transport of NSP4 to the PM, translocation across the PM, exposure of the C-terminus on the cell surface and subsequent secretion occurs via an unusual, complex and likely cell-dependent process. The exofacial exposure of the C-terminus poses several questions and suggests an atypical mechanism by which NSP4 traverses the PM and interacts with membrane lipids. Mechanistic details of the unconventional trafficking of NSP4, interactions with host-cell specific molecules and subsequent release require additional study.

## Background

Rotaviruses (RV), family reoviridae, are non-enveloped, triple-layered (VP2, VP6, VP7) virions with a single spike protein (VP4) and a genome of 11 double-stranded, RNA segments [1-3]. Six structural and six non-structural proteins are encoded by the segmented RNA genome. RV are the major etiological agent of severe, life-threatening gastroenteritis affecting 70% of young children worldwide [4,5]. Induction of RV diarrhea is multi-faceted involving several viral and host factors that contribute to the severity of disease (reviewed in [6-9]). RV fluid loss has been attributed to both an early, secretory diarrhea and a subsequent malabsorptive, hyper-secretive diarrhea due to the loss of absorptive enterocytes. The early secretory diarrhea likely is induced by NSP4 [9-12], which binds an integrin receptor and promotes calcium mobilization followed by a chloride secretory response [8,10,11,13,14]. Several hypotheses have been posed to explain the interactions of NSP4 with host cell exofacial molecules that result in fluid loss. The prevailing theory is that NSP4 is released from infected cells to interact with surface receptors of neighboring cells to trigger a specific signaling event that results in secretion.

NSP4 is encoded by RV gene 10 and organized into three N-terminal hydrophobic domains and a single, extended, C-terminal region. The primary translation product of 175 amino acids (aa) with an apparent molecular weight (*M*r) of 20 kD is co-translationally glycosylated to 29 kD and processed to the mature 28 kD NSP4 glycoprotein, but oligosaccharide processing does not proceed past mannose 8 [15,16]. NSP4 traverses the endoplasmic reticulum (ER) bilayer once (aa 22-44) such that the N-terminal 21 residues localize to the lumen of the ER and the remainder of the molecule (aa 45-175) extends into the cell cytosol [17-19]. Two *N*-linked, high mannose glycosylation sites are located within the short ER luminal domain at residues 8 and 18 [17]. These glycan moieties are sensitive to Endo-β-N-acetylglucosaminidase H (EndoH) digestion, which supports the lack of exposure to Golgi enzymes [20,21]. Additional support for the lack of Golgi processing includes NSP4 insensitivity to brefeldin A [22-24].

Localized within the NSP4 extended cytoplasmic domain is an amphipathic, α-helical region that folds as a coiled-coil (aa 93-137) and overlaps the enterotoxic and oligomerization domains, as well as several cellular and viral protein binding sites [19,25-30]. Circular dichroism (CD) and cross-linking analyses show NSP4 primarily oligomerizes into tetramers [19,31,32]. Crystallographic data of NSP4 95-137 confirm a coiled-coil structure that folds as a homotetramer with a buried cation, presumably calcium [26]. Relevance of this structure is not clear, however it has been postulated that the NSP4 enterotoxic activity is structure-related rather than sequence-specific [31,33,34].

NSP4 is a multi-functional glycoprotein that contributes to RV morphogenesis [19,35], replication [36] and pathogenesis [6,8,9,33,37]. At the ER, NSP4 functions as an intracellular receptor for double layered particles (DLPs) to facilitate the addition of the RV outer coat protein (VP7) in the ER and possibly the spike protein (VP4) [19,38-40]. RNA silencing (siRNA) studies of NSP4 expression reveal that in the absence of NSP4, there is: (i) an abnormal distribution of viral proteins in the viroplasm; (ii) little to no infectious viral particles in the cell, (iii) an accumulation of empty viral particles, and (iv) an increase of viral transcripts [35,36].

It has been established by several groups that exogenous introduction of NSP4 or the NSP4_114-135 _enterotoxic peptide from distinct genotypes with divergent sequences induces an age-dependent diarrhea in rodents [10,37,41,42] and may contribute to RV pathogenesis in other organs [43]. These data indicate NSP4 has distinct roles on opposite sides of the plasma membrane (PM) similar to select host cell proteins that function differently in intracellular and extracellular microenvironments [44,45].

NSP4 has been localized to multiple intracellular sites. A few examples include radiating from the ERGIC along microtubules into the cytoplasm [30], association with autophagosomes [22], bound to caveolin-1 (cav-1) [24,29,46,47], and in PM-derived caveolae [48]. Previous reports of NSP4 association with rafts suggest these lipid microdomains may contribute to secretion of NSP4 from infected cells [49,50]. Other studies show that the secretion of NSP4 into culture media is either a cleavage product [23] or an over-glycosylated form of NSP4 (~32 kD) [51]. Both of these studies indicate the release of NSP4 is dependent on secondary processing of the mature, 28 kD form.

Our previous data reveal that the native glycosylated, 28 kD NSP4 is distributed throughout the cell, including the cell periphery, and is present in enriched, PM-derived caveolae fractions that lack ER markers [24,29,48]. Herein we address the presence of NSP4 at the exofacial surface, the glycosylated form of NSP4 secreted from infected cells, and the directional transport of NSP4 in polarized cells of different origin. The temporal appearance of NSP4 at the exofacial PM and release into culture media in canine kidney (MDCK) and cloned human intestinal (HT29.F8) [52] cells were examined at specified times post RV infection and at a relatively low MOI. The results signify NSP4 intracellular transport and release from the cell is cell type-, viral strain-, and/or MOI-dependent.

## Materials and methods

### Antibodies and Reagents

NSP4_120-147 _and NSP4_150-175 _peptide-specific antisera were generated in rabbits and mice as previously described [10]. Peptide-specific rabbit antibodies were affinity purified with pre-activated cyanogen bromide Sepharose 4B beads to which the peptide was bound (Amersham Pharmacia Biotech Piscataway, NJ). NSP4 peptide-specific IgG was eluted by altering the pH [53,54] and reactivity confirmed by Western blot. NSP4_150-175_-specific F(ab)_2 _fragments were produced from the peptide-specific rabbit IgG using the ImmunoPure preparation kit (Pierce, Rockford, IL) [55] and conjugated to CY5 and/or CY3 monofunctional reactive dyes as described by the manufacturer (Amersham Pharmacia Biotech). Reactivity of the fluorescently-conjugated F(ab)_2 _was confirmed by immunofluorescent microscopy. Rabbit anti-NSP5 and guinea pig anti-NSP5 were the gifts of Dr. S. Lopez (Institute of Biotechnology, Morelos, Mexico) and Dr. O. Burrone (International Center for Genetic Engineering and Biotechnology, Trieste, Italy), respectively.

Rabbit anti-giantin (Convance Research Products, Inc., Princeton, NJ), mouse anti-sheep sodium/potassium (Na^+^/K^+^)-ATPase α (Affinity BioReagents, Inc., Golden, CO), rabbit anti-cav-1 (Cell Signaling, Danvers, MA), goat anti-rabbit IgG conjugated to horseradish peroxidase (HRPO), and goat anti-mouse IgG-HRPO (Southern Biotech Assoc, Inc, Birmingham, AL, or Pierce, Rockford, IL) were obtained from the indicated commercial sources. Linked fluorescent antibodies and molecules included Alexa Fluor 350-, CY3-, or CY5-conjugated streptavidin (Molecular Probes, Eugene, OR), goat anti-mouse IgG-Texas Red (Rockland Immunochem, Inc, Gilbertsville, PA), goat anti-mouse IgG-CY5, and the F(ab)_2 _fragment of goat anti-rabbit antibody-CY2 (Jackson ImmunoResearch, West Grove, PA).

EZ-Link^® ^Sulfo-NHS-SS-Biotin and streptavidin-agarose (Pierce), endo-β-N-acetylglucosaminidase H (EndoH; New England BioLabs, Ipswich, MA), protease inhibitor cocktail set III (Calbiochem, Darmstadt, Germany), and nucleofectin transfection reagent (Amaxa Biosystems, Cologne, Germany) were purchased from the indicated commercial sources.

### Cell Lines, Virus, and Viral Growth Curves

MA104 and MDCK cell lines were acquired from the American Type Culture Collection (Manassas, VA). The HT29.F8 cells, a spontaneously polarizing cell line, was derived from the parent human adenocarcinoma (HT29) intestinal line, grown in Dulbecco's modified minimal essential media (DMEM) and supplemented as previously described [52]. BHK-21 cells (gift of Dr. J. Leibowitz, TX Health Science Center, College Station, TX) were grown in Minimal Essential Medium (MEM; Mediatech) supplemented with 20 mM L-glutamine, 1 mM sodium pyruvate, 5% fetal bovine sera, 5% Serum Supreme, 100 U/L penicillin, 100 μg/L streptomycin, 0.25 μg/L Fungizone (Cambrex Corporation, East Rutherford, NJ). BHK-21 transfection media also included 10% tryptose phosphate broth and 10 mM 4-(2-hydroxyethyl)-1-piperazineethanesulfonic acid (HEPES, Amresco, Solon, OH), pH 7.3 ± 0.1.

RV SA11 clone 4F (SA11.4F, gift of Dr. M. Estes, Baylor College of Medicine, Houston, TX) was grown and titered in MA104 cells, and stored at -80°C. Titers were determined by indirect immunofluorescent staining of MA104 monolayers inoculated with serial dilutions of the virus and expressed as the number of focus forming units (FFU) per ml [56,57].

For RV infection, MDCK and HT29.F8 cell monolayers (~80% confluent) were starved for sera for 12-16 h prior to addition of virus as previously reported [29]. Briefly, the RV stock was sonicated (5 min using a cuphorn attachment ice bath in a Misonix Sonicator 3000; Misonix, Inc, Farmingdale, NY) and incubated in serum-free DMEM with 1 μg/ml trypsin (Worthington Biochemical, Lakewood, NJ) for 30 min at 37°C. The activated viral inoculum was added to the cells for 1 h at 37°C in 5% CO_2 _at an MOI of 2. The inoculum was replaced with serum-free DMEM supplemented with 1 μg/ml trypsin and incubated for various times post infection. For imaging, cells were grown to 40% confluency so that individual cells could be analyzed independently.

To evaluate the directional transport of NSP4 in the polarized kidney and intestinal cell lines, the cells were seeded at a density of 1 × 10^5 ^cells/cm^2 ^on Transwell permeable membrane supports (Corning Costar, Acton, MA) and separately on plastic at the same density to monitor confluency and the formation of domes, which are indicative of directional transepithelial water and electrolyte transport [58,59]. Transepithelial resistance (TER) was monitored prior to, during and post RV infection using a Millicel-ERS volt-ohmmeter according to the manufacturer's instructions (Millipore, Billerica, MA). The cells were infected with RV SAll.4F at a MOI of 2 only on the apical surface and maintenance of the polarized phenotype was monitored by TER.

To generate RV growth curves, MDCK and HT29.F8 cells were grown to 80% confluency in 35 mm tissue culture plates, starved for fetal bovine sera 12 h prior to infection with trypsin-activated RV SA11.4F at 37°C for 1 h at an MOI of 2 (as above). At specified times post infection, the media only or a combination of the cells and media were collected for RV titration. Media was clarified at 850 xg for 5 min and was stored at -80°C. Cells and media were collected, subjected to repeated freeze-thaws and stored at -80°C. Titers were determined by inoculating MA104 cells with serial dilutions of the media or the cells and media, and expressed as FFU/ml as reported [56].

### Transfection of pcDNA3.2DestNSP4 plasmid DNA

Nucleofector Solution L (Amaxa Biosystems) was used for transient transfection of pcDNA3.2DestNSP4 plasmid DNA in BHK-21 cells. The Amaxa Nucleofector II^® ^and Nucleofector I^® ^kits were employed following the manufacturer protocols (Amaxa Biosystems). BHK-21 cells were grown to 60-70% confluency (~2 × 10^6^/cm^2^), harvested, counted, and diluted to 1 × 10^6 ^cells per 100 μl. The cell suspension was mixed with 100 μl of cell-type specific Nucleofector solution L and 2 μg of pcDNA3.2DNSP4_1-175 _plasmid DNA using the Nucleofector program A-031. Transfected cells were plated onto 10 mm cover slips and incubated at 37°C in 5% CO_2 _for 20 h prior to image analyses. Alternatively, the transfected cells were plated onto 6-well plates and incubated at 37°C in 5% CO_2 _for 20 h prior to surface biotinylation and streptavidin pull down (below).

### Surface biotinylation and recovery of exofacial proteins

Cells were grown in 6- or 12-well culture plates, RV-infected at MOI of 2 or NSP4-transfected (above), and washed with ice-cold PBS-CM (PBS supplemented with 0.1 mM CaCl_2 _and 1 mM MgCl_2_) immediately before the addition of 0.5 mg/ml solution of Sulfo-NHS-SS-Biotin in ice-cold PBS-CM as previously described with minor modifications [59,60]. Infected, uninfected, transfected, or naïve cells to which NSP4-containing media had been added were incubated for 30 min at 4°C with cold NHS-S-S-Biotin solution. Excess biotin was quenched with an equal volume of cold DMEM with 10% bovine sera. Cells were treated with SDS-free RIPA buffer (150 mM NaCl, 50 mM Tris-base, 10% NP40, 0.5% DOC, pH 8.0) with protease inhibitors (protease inhibitor cocktail set III, Calbiochem) for 20 min at 4°C. Lysates were pelleted by centrifugation and the supernatants from equally treated cells were pooled. To extract the biotin-labeled surface proteins, 30 μl of streptavidin-agarose slurry (6% solution stock, Pierce) per ml of lysate was incubated at 4°C for approximately 14 h with constant rotation.

Streptavidin-agarose-bound proteins were pelleted at 12,000 xg for 20 min at 4°C and the supernatants carefully removed. The pelleted beads were washed with RIPA buffer, suspended in sample reducing buffer, boiled and loaded onto 12% SDS-PAGE gels for Western blot analyses (below). To ensure the lack of non-specific binding to the agarose beads, mock biotinylated (lacking NHS-SS-Biotin) and mock pull-down (lacking streptavidin-bound agarose) samples from both the RV- and mock-infected MDCK and HT29.F8 cells, or BHK-21 NSP4- and mock-transfected cells were included as controls. Cav-1, a cytofacial membrane protein [61-64] was used as a negative exofacial protein control.

Polarized cells grown on permeable supports were biotinylated at 24 hpi. NHS-SS-biotin solution was added to either the apical (100 μl) or basolateral (300 μl) surface. Media was added to the opposite surface that was not being biotinylated [60]. Care was taken to ensure the TER was maintained. All other steps of the biotinylation were the same as above.

### Live Cell Staining: Surface Antibody Binding

To control for biotin artifacts and show the presence of NSP4 at the exofacial surface by another technique, unfixed, RV-infected MDCK cells were reacted with rabbit anti-NSP4_150-175 _at 4°C and biotinylated rabbit-anti-IgG prior to cell lysis at 11 hours post infection (hpi), lysed in RIPA buffer, and precipitated with streptavidin agarose in the cold. Uninfected cells served as the control. The lysates and precipitated proteins were separated by 12% SDS-PAGE and probed with mouse anti-NSP4_114-135 _followed by anti-mouse IgG-HRPO by Western blot analyses.

### Protein Quantification

The micro bicinchoninic acid (BCA) protein assay (Pierce) was employed to quantify protein concentrations using bovine serum albumin as the standard per manufacturer's protocol.

### EndoH digestions

One μg aliquots of total protein from each sample and control were denatured and diluted per the manufacturer's protocol (New England Biolabs). As previously described, either 1-2 μl of sterile water (mock cleavage) or 1-2 μl of EndoH (2000 Units) were added to each sample or control and incubated at 37°C for 1 h prior to evaluation by Western blot.

### Western blot analyses

Western blots were performed to visualize (i) the presence of NSP4 on the cell surface when biotinylated, (ii) the relative size of the biotinylated, exofacial NSP4 molecule(s) at various times, (iii) the expression of NSP4 post RV-infection or pcDNA3.2DestNSP4-transfection, (iv) the potential shift in NSP4 mobility on SDS-PAGE following EndoH digestion, and (v) the preferential surface expression in polarized cells. Briefly, each sample was resolved by 12% SDS-PAGE and transferred to nitrocellulose filters according to the manufacturer (BioRad, Hercules, CA). The filters were blocked in 10% non fat dry milk (BLOTTO) in phosphate buffered saline, pH 7.2 (PBS) at 27°C for 1 h, and reacted with rabbit anti-NSP4_150-175 _diluted in 2.5% blotto/PBS (1:2000) for at 4°C 14 h. The membrane was washed in 0.5% BLOTTO/PBS with and without 0.05% Tween-20 (v/v), reacted with anti-rabbit IgG-HRPO (1:10,000) for 1 h at 27°C, washed again, and reacted with Western chemiluminescent substrate (Millipore, Bedford, MA) per the manufacturer's instructions. As indicated, substrate with femtogram sensitivity was substituted (Pierce) when necessary.

Once the cells polarized, apical and basolateral membranes were differentially biotinylated, an equal quantity of protein was separated by SDS-PAGE in separate lanes and examined by Western blot. Cell lysates and the biotinylated apical or basolateral surfaces were probed with the same antibodies as listed above.

### Laser Scanning Confocal Microscopy (LSCM)

Specific fluorescently-tagged IgG or F(ab)_2 _fragments were used with LSCM to visualize NSP4 and marker proteins in RV-infected cells. Cells were treated with antibodies post fixation and permeabilization to conduct fluorescent resonance energy transfer (FRET) analyses. The fluorescent images were captured with a MRC-124MP BioRad LSCM system (BioRad) using a Zeiss inverted Axiovert microscope (Carl Zeiss, Inc., Thronwood, NY), a 63X Zeiss oil apochromat objective, and the 488, 568 or 647 nm excitation lines of an argon/krypton ion laser source. LaserSharp 3.0 (BioRad), Confocal Assistant 4.02 (Brelje TC/BioRad), Metamorph 4.0 (Molecular Devices Corporation, Sunnyvale, CA), Image J (public domain Java image-processing program inspired by NIH Image), and Adobe Photoshop 7.0 (Adobe Systems Inc., San Jose, CA) were employed depending on the application to capture the pixilated PMT data, convert the image data to tiff format, and adjust for contrast curves to construct the final images.

RV SA11.4F- and mock- infected or NSP4- and mock-transfected cells were grown on glass cover slips, fixed and permeabilized with methanol acetone (1:1 vol/vol) for 5 min at -20°C prior to fluorescent staining at various hpi or at 20 h post transfection. Non-specific binding sites were blocked with 4% BLOTTO in PBS for 45 min at 27°C. Primary antibodies were diluted in 2% BLOTTO-PBS and incubated with the cells for 45 min at 27°C. Following a series of PBS washes, secondary antibodies (CY2-, Texas Red-, or CY5-conjugated, species-specific IgG) were incubated for 45 min in the dark. The PBS wash was repeated in the dark and the cover slips mounted onto glass slides with fluorescent mounting media (Kirkegaard & Perry Laboratories, Inc., West Chester, PA).

### FRET by acceptor photobleaching

To confirm the exofacial exposure of NSP4, FRET by acceptor photobleaching was employed with mock- and RV-infected cells [65,66]. Cells were surface biotinylated and probed with NSP4_150-175_-F(ab)_2_-CY 5 and streptavidin-CY3. A series of images were recorded both before and after (568 and 647 nm, 10% power) photobleaching (100% power for 3 min) of the CY5 fluorophore and the integrated density value (IDV) was determined. The unquenching of the CY3 donor signal (568 nm) was measured indicating FRET due to acceptor photobleaching (2 fluorophores are within 10 nm). For each time point, a minimum of 20 fields were analyzed from 2 independent experiments. The ratio F_DA_/F_D _was calculated, where F_DA _is the fluorescence intensity (pre-bleach) of the donor in the presence of the acceptor and F_D _is the fluorescence intensity (post-bleach) of the donor in the absence of acceptor. By measuring the IDV of the donor (CY3) signal before acceptor (CY5) photobleaching divided by the donor (CY3) IDV post bleach signal (i.e. CY3 IDV pre-photobleaching/CY3 IDV post), the amount of FRET was estimated. The change in IDV of the donor in the presence of acceptor was calculated as follows: CY3 IDV post photobleaching - CY3 IDV pre-photobleaching/CY3 IDV post. The % change in IDV due to a FRET interaction was calculated for every field that was photobleached and the average was calculated from 3 independent experiments for each time point. CY3-labeled surface proteins in a 500 by 100 pixel area outside of the photobleached region served as the negative control area for each analyzed cell within a given field of view. Image analyses were completed using Image J software. Since the amount of biotinylated proteins on the surface of the PM far exceeded the amount of surface-biotinylated NSP4, we employed an equation describing the non-interacting donors in combination with the fraction of donor-acceptor pairs that interact at a mean distance *r*, written as: *q*_*m *_= *f_u_q_u _*+ *f_b_q_b _*= (1 - *f_b_*) + *f_b_q_b _*and rearranged to . *q_m _*is the measured relative quantum yield resulting from FRET but containing background donor signal and also is the measured ratio of F_DA_/F_D; _*q_u _*is the quantum yield of the "unbound" donors not specifically involved in FRET that was normalized to 1; *f_u _*is the fraction of "unbound" donors not specifically involved in FRET equal to 1- *f_b_*; *q_b _*is the quantum yield (after the unbound donor quantum yield was normalized to 1) resulting from FRET of the donor-acceptor pairs; and *f_b _*is the fraction of donors that are "bound" to an acceptor [66,67]. Geometric constraints upon the position of the donor CY3 labeling the streptavidin and the position of the acceptor CY5 on the NSP4_150-175_-F(ab)_2_-CY5 complex provided an estimate of the mean FRET interaction radius. Since the radius of the streptavidin has been stated as approximately 27 Å [68,69] and the radius of the F(ab)_2 _as approximately 35 Å [70,71] a mean FRET interaction distance of 62Å was used. Using the two equations,  and *E *= 1 *- q_b _*, where *E *is the energy transfer due to FRET between a donor and acceptor pair, *R_0 _*is the Förster distance for fluorescent probes, CY3 and CY5, and *r *is the mean interaction distance between many donor and acceptor pairs resulting from the CY3-streptavidin-NSP4_150-175_-F(ab)_2_-CY5 complexes on the surface of the PM. Using *R_0 _*= 50Å and *r *= 62Å, the energy transfer was calculated to be *E *= 0.2157 and thus q_b _= 0.7843. These values were subsequently used to determine the % of fluorescent donors involved in FRET interactions with CY5 acceptors as shown in the Results.

### Epifluorescence of Transfected BHK-21 cells

BHK-21 cells were transfected with pcDNA3.2DestNSP4 using nucleofectin prior to colocalization of NSP4 and Na^+^/K^+^-ATPase (PM marker). Transfected and non-transfected cells were visualized using a Stallion Digital Imaging Workstation (Carl Zeiss) equipped with 300W xenon fluorescent light source with rapid switching (<2 msec) between excitation wavelengths. Images were collected using a 63X objective 0.75 numerical aperture, ROPER CoolSnap HQ camera and Slidebook 4.2 software (Intelligent Imaging Innovations, Denver, CO). The Stallion system filter sets include the excitation/emission wavelengths for: (i) FITC and CY2 (470/20:505-530 nm) to detect anti-rabbit IgG-CY2, (ii) Texas Red and CY3 (560/40:590 nm) to detect anti-mouse IgA-Texas Red and (iii) CY5 (600/50:685/50 nm) to detect streptavidin-CY5. All images, including the series of merged colocalizations were processed using Image J (NIH).

### Addition to naïve cells of RV-free, soluble NSP4 released into culture media of infected cells

Culture media from RV SA11.4F-infected or mock-infected cells were collected, clarified by centrifugation at 850 × g for 5 min to remove intact cells and cellular debris, followed by 92,000 × g to remove viral particles. The supernatants then were collected and tested for the presence of RV by FFU assays to rule out the presence of infectious virus. The presence of NSP4 was confirmed by immunoprecipitation [24]. The supernatants lacking RV were concentrated approximately 10-fold using an iCON™ Concentrator (Pierce, MWCO 9 kD) and diluted 6-fold in DMEM to evaluate the first time point. For the other time points, clarified media directly was reacted with uninfected MDCK or HT29.F8 cells for 1.5 h at 4°C to allow NSP4 attachment and avoid endocytosis. The cells were washed and surface biotinylated as described above. To detect the presence of NSP4 bound to the surface of the uninfected cells, the treated cells were lysed and the lysates were precipitated with streptavidin-agarose and both subjected to Western blot.

### Detection of released NSP4 on the surface of RV-infected HT29.F8 cells in culture

At 7 hpi, RV-infected HT29.F8 cells were surface biotinylated and fixed at 4°C. Streptavidin-CY2 was added to the fixed cells to label all exofacial proteins. NSP4 was reacted with CY5-linked NSP4_150-175 _F(ab)_2_. LSCM was utilized to detect infected cells via the presence of intracellular NSP4 and for the presence of NSP4 on the surface of neighboring, uninfected cells. Colocalization of the streptavidin-CY2 and NSP4-CY5 appeared yellow.

## Results

### Kinetics of NSP4 transport to the exofacial surface of MDCK and HT-29.F8 cells were distinct

**Figure 1:**
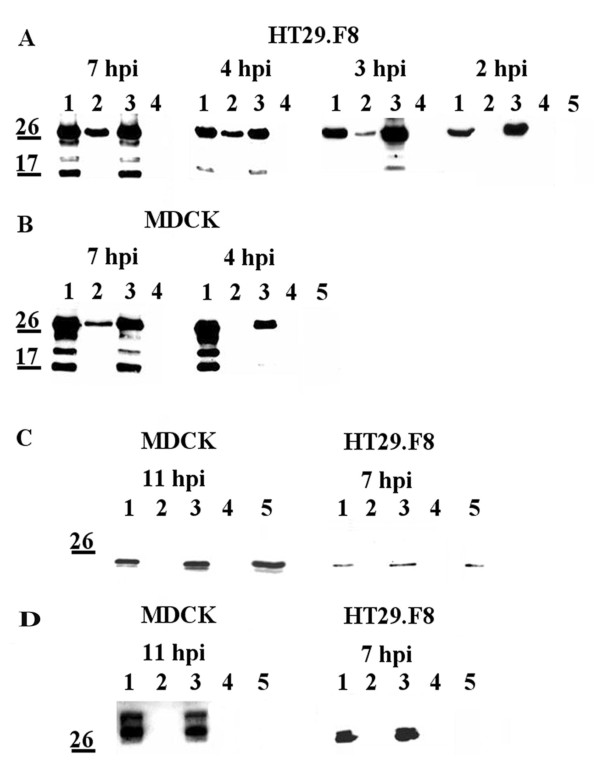
**NSP4 transport kinetics to the exofacial PM of HT29.F8 and MDCK cells vary**. The cloned intestinal and canine kidney cells simultaneously were infected with RV SA11.4F (MOI = 2). At 2, 3, 4, 7, and 8 hpi, the cells were surface biotinylated at 4°C, lysed, and precipitated with streptavidin agarose. The cell lysates and precipitated surface proteins were separated by SDS-PAGE and analyzed by Western blot using NSP4_150-175_.peptide-specific antibodies (panels A and B). A cytofacial membrane (anti-caveolin-1) and intracellular (anti-NSP5) control are shown in panels C and D, respectively. Lanes were loaded as follows: Lane 1 = cell lysates recovered from the streptavidin pull-downs. Lane 2 = streptavidin precipitate of the surface biotinylated proteins. Lane 3 = cell lysates from a mock-biotinylated control. Lane 4 = streptavidin precipitate of the mock-biotinylated control, Lane 5 = cell lysates from an uninfected cell control.

To delineate the temporal expression of NSP4 at the exofacial surface of MDCK and HT29.F8 cells, we evaluated the appearance of NSP4 on the cell surface by surface biotinylation followed by streptavidin pull-downs and Western blots. Initially the expression of NSP4 was established on the exofacial surface of both cell lines at 8, 11 and 24 hpi (not shown) and then was examined at sequentially earlier time points (7, 4, 3 and 2 hpi). We also tested different MOI to ensure a MOI of 2 yielded detectable expression of NSP4 in the absence of membrane disruption. Total cell lysates (Figure 1, **Panels A and B**, lanes 1 and 3) revealed the presence of NSP4 in intact cells using a MOI of 2. Lysates of both cell types showed the fully glycosylated, native 28 kD, the single glycosylated (26 kD) and the unglycosylated (20 kD) forms of NSP4 at 7 and 4 hpi. In addition, a band near 17 kDa consistently was noted in these lysates. In the HT29.F8 cells, streptavidin-bound, surface-exposed NSP4 was detected at 7, 4 and 3 hpi but not at 2 hpi (Figure 1A, lanes 2) indicating NSP4 had been transported to the exofacial membrane between 2 and 3 hpi in the human intestinal cell line. Only the full-length (FL), double glycosylated NSP4 was apparent in the precipitated, cell surface samples (Figure 1A, lanes 2). There consistently was a lack of the other forms of NSP4 and the 17kD band on the cell surface. Similarly, only the FL NSP4 (28 kD) was observed in the HT29.F8 lysates at 2 and 3 hpi (lanes 1). The identity of the 17 kD NSP4 band in infected lysates is unknown, but may correspond to the 15 kD product noted by Zhang, et. al, 2000 [23].

Examination of the MDCK cells similarly revealed the presence of FL NSP4 on the exofacial surface but only at 7 hpi (Figure 1B, 7 hpi, lane 2). At the next time point, 4 hpi, NSP4 was present in the lysates, but absent from the surface of the kidney cells (Figure 1B, 4 hpi, lanes1-2). Earlier time points were completed, but lacked NSP4 surface exposure and are not shown, n = 5. These data indicate NSP4 trafficked to the exofacial membrane of the MDCK cells between 4 and 7 hpi.

Controls included mock-biotinylated, RV-infected cells and the corresponding streptavidin pull downs (Figure 1, lanes 3 and 4, respectively) and an uninfected cell control (lanes 5). Two control antibodies, anti-cav-1 (cytofacial membrane control) (Figure 1C) and anti-NSP5 (cytosolic control) (Figure 1D) were employed to ensure the cells were intact when the biotin reagent was applied and that there was an absence of biotin entering the cell at the later time points. Cav-1, a protein known to traverse the cytofacial leaflet with both termini cytosolic in a hairpin structure [61-64], was absent from the surface of the infected cells at all time points (Figure 1C, lanes 2), MDCK cells at 11 hpi and HT29.F8 cells at 7 hpi. These times were selected because of the strong NSP4 signal routinely acquired in the respective cells and time. Similarly, NSP5 was absent from the exofacial PM (Figure 1D, lanes 2) at 7 (HT29.F8 cells) and 11 hpi (MDCK cells). Lanes 1 and 3 (Figure 1C-D) are the total cell lysates, which showed that the control proteins were expressed, but not exposed on the cell surface (lanes 2).

Because both cell lines simultaneously were infected with the same RV inoculum at an MOI of 2 and concurrently evaluated, these data suggest the difference in the transport kinetics and surface exposure of NSP4 was due to variation in the cells and how NSP4 interacts with specific host-cell molecules, not the virus or the conditions during the infection. Surface exposure of NSP4 at the same time NSP5 and cav-1 remained intracellular indicated the PM was intact in the same cells at the same hpi.

### Detection of the NSP4 C-terminus on the exofacial membrane by live cell staining and the lack of biotin artifacts

**Figure 2:**
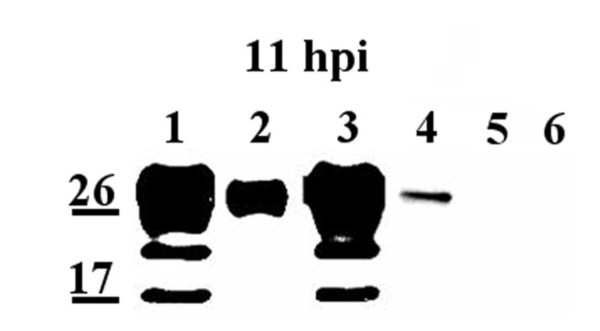
**NSP4_150-175 _peptide-specific antibodies were bound to the surface of live, RV-infected MDCK cells at 4°C**. MDCK cells were infected with RV SA11.4F at a MOI of 2 for 11 h and either surface biotinylated followed by precipitation with streptavidin agarose (lane 2) or reacted with rabbit affinity-purified anti-NSP4_150-175 _and biotin-conjugated anti-rabbit IgG, then similarly precipitated with streptavidin agarose (lane 4). Cell lysates and the precipitated surface proteins were examined by Western blot using mouse anti-NSP4_114-135_. Lanes 1 and 3 are the cell lysates from the streptavidin pull-down and antibody binding, respectively. Lanes 2 and 4 show the full-length NSP4 exposed on exofacial surfaces, and lanes 5 and 6 are uninfected controls.

To control for biotin artifacts, uninfected and infected MDCK cells (MOI = 2, 11 hpi) were either biotinylated or reacted with a NSP4 C-terminal specific antibody, anti-NSP4_150-175_, and a biotinylated anti-rabbit IgG in the cold (Figure 2). Streptavidin-beads were added to the biotinylated or biotin antibody-treated cells prior to cell lysis. The pelleted surface proteins and supernatants were examined by Western blot. Lysates of the infected cells (Figure 2, lanes 1 and 3) showed the same NSP4 banding pattern previously observed in the MDCK and HT29.F8 cells. The biotinylated-(Figure 2, lane 2) and the NSP4 peptide antibody-bound precipitates (Figure 2, lane 4) showed a single NSP4 band at 28 kD supporting the exposure of the C-terminus of FL NSP4. Antibody and biotin pull-down controls of uninfected cells are shown in Figure 2, lanes 5 and 6, respectively. Comparison of the band intensities showed that NSP4 more efficiently bound biotin (lane 2) when compared to binding the C-terminal antibody (lane 4). This may reflect a difference in detection sensitivity due to the ability of the small biotin molecule to bind multiple sites on the exposed NSP4 C-terminal domain whereas the peptide antibody may have limited access to a single epitope at the C-terminus. Although less efficient, a single 28 kD band was observed when the NSP4_150-175 _-specific IgG were bound to the cell surface that appeared similar to that seen with the biotinylated cells. These data indicate a lack of biotin artifacts and confirm the presence of at least the NSP4 C-terminus on the cell surface.

### FRET by acceptor photobleaching confirms the exofacial localization of NSP4

**Figure 3:**
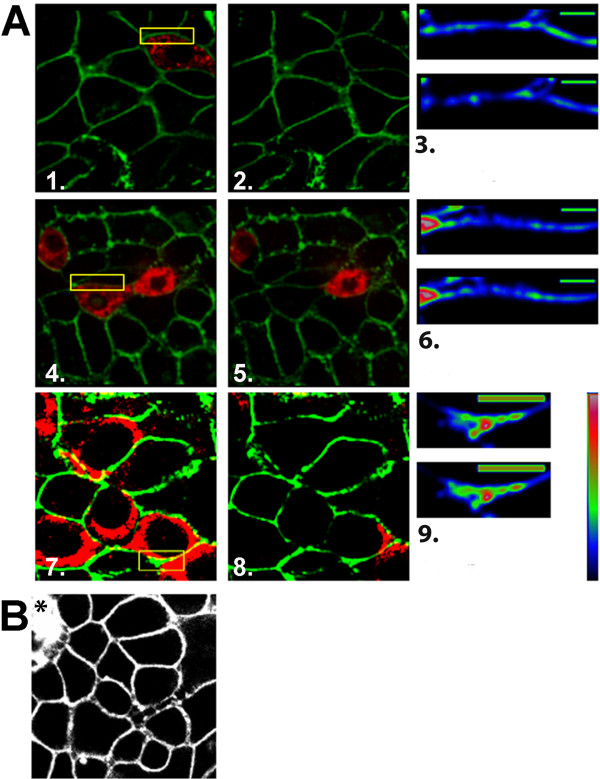
**Positive FRET analyses by acceptor photobleaching between NSP4 and surface proteins of RV-infected MDCK and HT29.F8 cells**. Live cells were surface biotinylated prior to fixation and permeabilization. Streptavidin-CY3 was utilized to label all surface biotinylated proteins of the exofacial PM including NSP4, if exposed. NSP4 was labeled with Cy5-linked F(ab)_2 _prepared from NSP4_150-175 _rabbit antisera. **A**. CY5 (NSP4) and CY3 (PM) images were acquired with the 647-nm and 568-nm-wavelength lasers both prior (merged CY3 and CY5 images, panels 1,4,7) and after (same cells, panels 2,5,8) photobleaching of the NSP4 CY5 (donor) signal in infected MDCK cells at 4 hpi (panels 1,2,3) and 7 hpi (panels 4,5,6). RV-infected HT29.F8 cells (MOI = 2) were examined at 7 hpi (panels 7,8,9). FRET was calculated from the fluorescence intensity of selected areas of the PM (yellow box) both before and after bleaching. Panels 3, 6, 9 show a magnification of the photobleached regions of the PM delineated by the yellow boxes in panels 1, 4, and 7. Panels were pseudo-colored to show relative intensities with its respective calculated FRET values. Both the pre (top panels of 3, 6, 9) and post bleaching (bottom panels 3, 6, 9) images are shown. Values were calculated from 10 different regions taken from two separate experiments for each time point. Controls for each region were calculated from a region of PM in which NSP4 was not present and was calculated at 0% FRET. Vertical bar = relative intensity scale with red > green > blue. **B **shows an example of a cell that lacked membrane integrity (*) and therefore intracellular molecules were biotinylated and bound CY3. Cells showing intracellular staining with CY3 were not used.

Because of the enhanced sensitivity of FRET analyses, FRET by acceptor photobleaching was employed using the CY5/CY3 fluorophore pairs (detection within 10 nm) to confirm NSP4 at the exofacial PM on individual cells while simultaneously analyzing the PM integrity (Figure 3). Analyses were limited to cells that appeared to be intact by monitoring the stained PM of the biotinylated, CY3 labeled cells by LSCM. When the membrane lacked integrity, intracellular proteins were biotinylated and bound CY3 (Figure 3B) and were not utilized in the study.

Two time points were selected for examination, one in which NSP4 was not detected on the exofacial membrane of RV-infected MDCK cells by surface biotinylation (4 hpi) and a time when NSP4 was observed at the exofacial PM (7 hpi) in both MDCK and HT29.F8 cells. The photo-acceptor fluorophore, CY5, was photobleached while the intensity of the donor fluorophore (CY3) was assessed. In this way, the quantity of the unquenched donor intensity due to release from FRET between the donor and acceptor pairs post photobleaching was measured. Figure 3A, panels 1, 4 and 7 show the merged images prior to photobleaching; panels 2, 5 and 8 show the merged images immediately post bleaching; and panels 3, 6, and 9 are magnified images of the green pseudo colored CY3-PM within the outlined yellow regions in panels 1, 4 and 7, both pre (top image) and post (lower image) photobleaching. At 4 hpi in MDCK cells, there is an absence of FRET; the integrated intensity was similar to that of the negative control with the F_DA_/_FD _ratio normalized to 1 or a 0% change in the integrated intensity (Figure 3A, panels 1-3, n = 3). These data confirmed the lack of NSP4 at the surface of MDCK cells at 4 hpi. At 7 hpi, a positive FRET reaction as determined by the % change of the IDV of the donor CY3 in the DA pair was noted between NSP4 and the biotinylated exofacial PM proteins in the examined area (yellow box, Figure 3A, panels 4-6). The % change in IDV in the MDCK cells at 7 hpi ranged from 3.2-6.8% with an average of +3.9% (panel 6, n = 20). Using the equations defined in the Methods, this implied that 18.1% of fluorescent CY3 labels were involved in a FRET interaction with the CY5 acceptor. HT29.F8 cells similarly were treated at 7 hpi and served as the positive control (panels 7-9). The average % change in IDV in the HT29.F8 cells that was gained due to photobleaching was +4.5% (panel 9, n = 10). Approximately 20.9% of fluorescent CY3-PM labels were involved in a FRET interaction with the CY5-NSP4 acceptors in the HT29.F8 membranes within the yellow box. Negative controls for each region were calculated from a section of the PM in which NSP4 was not present. As an additional control, CY5-cav-1 IgG was analyzed with CY3-streptavidin following surface biotinylation at 7 hpi and yielded 0% change in IDV implying no FRET (data not shown). Taken together, the positive FRET reaction between the biotinylated surface proteins bound by CY3-streptavidin and NSP4 bound by Cy-5-NSP4_150-175 _F(ab)_2 _in RV-infected MDCK cells at 7 hpi, but not at 4 hpi, confirmed our biotinylation/pull down kinetics results. As anticipated, the % change in IDV of NSP4 and exofacial molecules in the HT29.F8 cells was positive (Figure 3A).

Given that the Cy-5-labelled (Fab)_2 _fragments were prepared from NSP4_150-175 _IgG, these data indicate at least the C-terminus of NSP4 was exposed/available for antibody and/or biotin binding on the cell surface. These experiments were repeated using anti-NSP4_120-147_, which also is within the NSP4 C-terminal region, and the FRET reactions were positive with a similar average % change in IDV (data not shown). Efforts to prepare high-titer antibodies to the hydrophobic N-terminus, NSP4_2-22_, were not successful so we were unable to test the relative intracellular location of the N-terminus.

### Surface exposed NSP4 was EndoH sensitive early post infection

**Figure 4:**
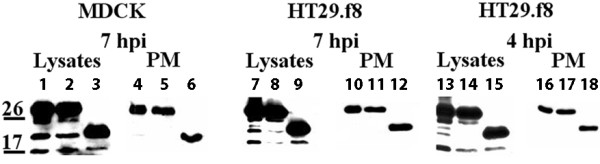
**EndoH sensitivity of NSP4 localized to the exofacial surface**. MDCK and HT29.F8 cells were infected with RV SA11.4F for 7 h (MOI = 2) biotinylated at 4°C, lysed, and treated with EndoH prior to Western blot analyses. Lysates and precipitated surface proteins from MDCK cells at 7 hpi and from HT29.F8 cells at 7 and 4 hpi are shown. Lane: 1 = MDCK lysates, pre EndoH treatment, 7 hpi. 2 = MDCK lysates, mock EndoH treatment, 7 hpi. 3 = MDCK lysates, EndoH treated, 7 hpi. 4 = MDCK PM (streptavidin pull-down), pre Endo H treatment, 7 hpi. 5 = MDCK PM, mock EndoH treatment, 7 hpi. 6 = MDCK PM, Endo H treated, 7 hpi. 7 = HT29.F8 lysates, pre EndoH treatment, 7 hpi. 8 = HT29.F8 lysates, mock EndoH treatment, 7 hpi. 9 = HT29.F8 lysates, EndoH treated, 7 hpi. 10 = HT29.F8 PM (streptavidin pull-down), pre EndoH treatment, 7 hpi. 11 = HT29.F8 PM, mock EndoH treatment, 7 hpi. 12 = HT29.F8 PM, EndoH treated, 7 hpi. 13 = HT29.F8 lysates, pre EndoH treatment, 4 hpi. 14 = HT29.F8 lysates, mock EndoH treatment, 4 hpi. 15 = HT29.F8 lysates, EndoH treated, 4 hpi. 16 = HT29.F8 PM, pre EndoH treatment, 4 hpi. 17 = HT29.F8 PM, mock EndoH treatment, 4 hpi. 18 = HT29.F8 PM, EndoH treated, 4 hpi.

To determine the glycosylation state of the FL NSP4 molecules on the exofacial PM early post infection, the RV-infected MDCK and HT29.F8 cells were surface biotinylated, precipitated with streptavidin agarose, mock or EndoH digested, and evaluated by Western blot. Seven hpi was examined to ensure NSP4 surface exposure in both cell types (Figure 4). Akin to that observed in the kinetics experiment (above) and the polarity study (below), the infected cell lysates contained the 2 glycosylated forms (28 and 26 kD) and the unglycosylated form (20 kD) of NSP4, and a band near the 17 kD marker when mock-glycosidase treated or immediately prior to EndoH treatment (Figure 4, lysates, lanes 1-2, 7-8 and 13-14). EndoH digested lysates revealed a shift in NSP4 electrophoretic mobility with the 28 kD double-glycosylated band shifting to about 20 kD in both cell lines (Figure 4, lysates, lanes 3, 9 and 15, n = 5). There was no obvious change in the mobility of the unglycosylated 20 or the 17 kD bands.

When the precipitated, surface-exposed fractions were examined by Western blot (PM fractions), the doubly-glycosylated (28 kD) form of NSP4 was the only form detected prior to EndoH digestion (Figure 4, PM, lanes 4-5, 10-11, and 16-17, n = 5) except for a very faint 20 kD band in lane 4. EndoH treatment of the surface-exposed NSP4 resulted in a shift from the 28 kD protein the 20 kD unglycosylated form (Figure 4, PM, lanes 6, 12 and 18) in both cell lines. These data support our previous results showing that NSP4 in isolated caveolae from enriched PM fractions is EndoH sensitive [48] and verifies that the 17 kD protein bands lack the N-terminal glycosylation sites. We also evaluated the EndoH sensitivity of NSP4 at the surface of HT.29F8 cells at 4 hpi (Figure 4). These data agreed with the HT29.F8 kinetic data showing only the fully-glycosylated protein was exposed on the cell surface at 4 hpi and also revealed it was EndoH sensitive at this early time point.

### NSP4 directional transport in polarized cells

**Figure 5:**
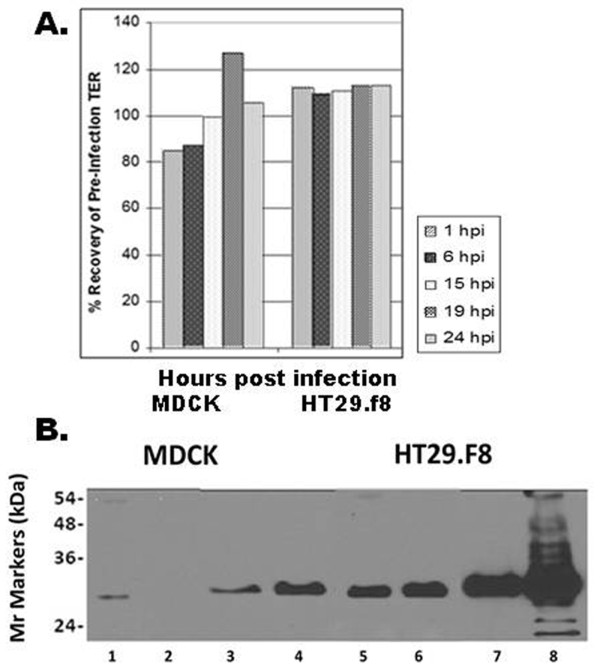
**Preferential sorting of NSP4 in polarized kidney and intestinal cells was distinct**. MDCK and HT29.F8 cells were grown on permeable, membrane supports until differentiation as indicated by an elevated transepithelial resistance (TER, ≥ 500 ohms × cm^2^) and the presence of domes in cells grown on plastic. The cells were simultaneously infected with RV SA11.4F on apical membranes at MOI = 2 and the TER monitored to ensure the polarized phenotype was maintained 1 - 24 hpi. Shown in **Panel A **are the TER measurements of the infected MDCK and HT29.F8 cells taken at 1, 6, 15, 19, and 24 hpi. The x-axis indicates the hpi and the y-axis shows the corresponding % recovery of the pre-infected TER. **Panel B**. Apical or basolateral surfaces were biotinylated at 24 hpi with opposite surfaces treated with media. The cells were lysed, precipitated with streptavidin-agarose and subject to SDS-PAGE and Western Blot (Panel B). The *M*r markers are shown on the left. Lanes 1-4 are from the MDCK cells and lanes 5-8 from the HT29.F8 cells, with lanes 1 and 5 showing the apical surface, and lanes 2 and 6 the basolateral surfaces. Lanes 3-4 and 7-8 are the corresponding cell lysates.

To monitor the directional transport of NSP4 in polarized epithelium, MDCK and HT29.F8 cells were grown on permeable supports until a high TER (≥500 ohms × cm^2^) was reached and then virus was added to apical membranes at a MOI of 2. As expected, there was an initial drop in the resistance immediately post infection that completely recovered by 1 hpi in the HT29.F8 cells and 15 hpi in the MDCK cells to pre-infections levels (Figure 5A). The MDCK cells displayed ~80% of the pre-infection TER at 1 and 6 hpi. By 19 hpi, the TER values in the MDCK cells increased to greater than 120% of the pre-infected values (Figure 5A). Maximal TER values typically ran at or greater than 600 ohms × cm^2 ^with the HT29.F8 displaying higher overall TER values as previously seen [52]. Several MOI's were examined (not shown) and a MOI of 2 was found to yield a detectable level of NSP4 while maintaining the polarized phenotype.

At 24 hpi, cells were surface biotinylated on apical or basolateral surfaces, precipitated with streptavidin-agarose, and evaluated by Western blot as before (Figure 5B). In MDCK cells, NSP4 only was detected on apical membranes (Figure 5B, lane 1) and was absent from basolateral membranes (lane 2) indicating NSP4 primarily was transported to the apical surface in the kidney cells under the indicated test conditions. Cell lysates are shown corresponding to the biotinylated apical (lane 3) and basolateral (lane 4) MDCK surfaces. In contrast, NSP4 was detected on both the apical (lane 5) and basolateral (lane 6) surfaces of the HT29.F8 cells under the same test conditions. The corresponding intestinal cell lysates are shown in lanes 7 and 8. Similar to that seen in the kinetics study, the level of NSP4 expression appeared higher in the HT29.F8 cells when compared to that of the MDCK cells (Figure 5B, cell lysates, lanes 3, 4 vs. lanes 7, 8; and on membranes, lanes 1-2 vs. 5-6) even when infected at the same MOI under the same conditions and when the samples consistently were loaded onto SDS-PAGE gels at equivalent ug/lane. There was a barely visible band near the 54 kD marker in lane 1 (MDCK apical surface), which presumably is a dimer of the 28 kD NSP4. It is unclear why only the HT29.F8 lysates that were biotinylated on the basolateral surface showed multiple glycosylated forms of NSP4 (lane 8) or, alternatively, why the other lysates only showed the double-glycosylated form. Finally, the preferential transport of NSP4 to distinct membranes in different cell types again implies unique interactions of NSP4 with specific host-cell molecules.

### Released NSP4 was detected in culture media at 6-8 hpi

**Figure 6:**
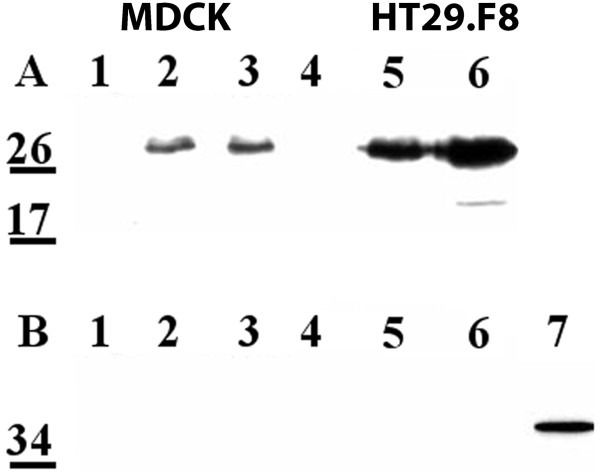
**NSP4 was detected in the media of intact, RV-infected MDCK and HT29.F8 cells**. Cells were infected with RV SA11.4F at a MOI of 2 for 6, 8 and 12 hpi. At each time point, the media was collected, clarified to remove cell debris, and ultra-centrifuged to remove RV particles prior to separation by SDS-PAGE and analyses by Western blot. **Panel A: **membrane was probed with anti-NSP4_150-175_. The clarified media collected at 6 hpi from MDCK (lane 1) or HT29.F8 (lane 4) cells were concentrated 10-fold prior to analyses. The clarified media collected at 8 and 12 hpi from RV-infected MDCK cells (lanes 2 and 3, respectively) and HT29.F8 cells (lanes 5 and 6, respectively) were analyzed in the absence of concentration. **Panel B**: the same membrane was probed with anti-GAPDH as a membrane integrity control. Lane 7 is HT29.F8 cell lysates showing the presence of GAPDH (positive antibody control).

Once we established that FL NSP4 was exposed on the exofacial PM of RV-infected cells, we then evaluated the extent of NSP4 release into culture media at 2-12 hpi while ensuring that the infected cells were intact by probing for the cytosolic enzyme, d-glyceraldehyde-3-phosphate dehydrogenase (GADPH). At each time point, the media from both cell lines were removed and clarified at 192,000 × g for 3 h to ensure the absence of virus (verified by the lack of FFU). The media that was collected and analyzed at 2 and 6 hpi were concentrated about ten times to enhance the detection of small amounts of secreted NSP4. Whereas the media collected at 8 and 12 hpi were clarified and directly evaluated in the absence of concentration. At 2 hpi (not shown) and 6 hpi (Figure 6A, lanes 1 and 4), NSP4 were not detected in the concentrated media of either cell line. In contrast, a single, 28 kD NSP4 band was detected in the media at 8 and 12 hpi both from the MDCK (lanes 2 and 3) and HT29.F8 cells (lanes 5 and 6). Cells were simultaneously probed for GADPH as a cytosolic, membrane integrity control (Figure 6B). Only lane 7 (lysates) showed positive GADPH reactivity with anti-GADPH. The lack of NSP4 at 6 hpi (MOI = 2) and the presence of NSP4 at 8 hpi in media of both the MDCK and HT29.F8 cultures indicate that NSP4 was released from both the intestinal and kidney cells between 6-8 hpi while the cells were intact (lack of GADPH). There appeared to be a stronger signal for NSP4 in the media of the HT29.F8 cells (Figure 6A, lanes 5 and 6) when compared to an equal volume of media from the MDCK cells (lanes 2 and 3). At 12 hpi, there was a small amount of the unglycosylated NSP4 in the media of the intestinal cells only. It is unclear if this was a specific release or if some of the cells were becoming leaky at this time point, even though the stain for GADPH remained negative.

### RV growth curves showed virus release from both the HT9.F8 and MDCK cells between 6-8 hpi

**Figure 7:**
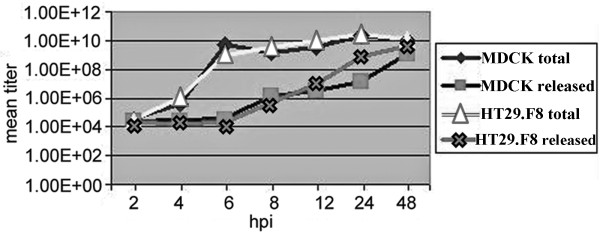
**RV SA11.4F growth curves in MDCK and HT29.F8 cells**. The kidney and intestinal cell lines were RV infected at MOI = 2. At 2 to 48 hpi, the media was collected to assess for released virus or cells and media were collected to evaluate total virus production. Samples were titered for RV by determining the FFU/ml. The average of 3 different assays for each time point is graphically shown.

Our data showing the release of NSP4 at similar times (6-8 hpi) in both the human, cloned intestinal and canine kidney cell lines were surprising given the difference in intracellular transport kinetics to the cell surface. To evaluate a possible role of viral replication and release in the secretion of NSP4, RV growth curves were generated in both cell lines (Figure 7, n = 3). Culture media (to monitor virus release) or cells and media (to evaluate total virus production) were collected at 2-48 hpi and titered by measuring FFU/ml. The growth curve of RV SA11.4F in MDCK cells resembled that previously reported [72] (diamonds and squares) and the RV growth curve in the HT29.F8 cells (triangles and outlined x) was similar to that of the MDCK cells. The curves were generated by averaging the RV titer from the media only or from the cells plus media at various hpi (n = 3, P < 0.5). Although the difference in NSP4 transport kinetics to the cell surface (2-3 hpi for HT29.F8 cells; 4-7 hpi in MDCK cells) did not appear to be due to differences in RV replication, it is noteworthy that the growth curves showed that virus was released from both cell types (6-8 hpi) at about the same time NSP4 was released (6-8 hpi). These data suggest that the release of RV into media may have influenced the secretion of NSP4 in both cell lines.

### NSP4 traffics to the PM in the absence of other viral proteins

**Figure 8:**
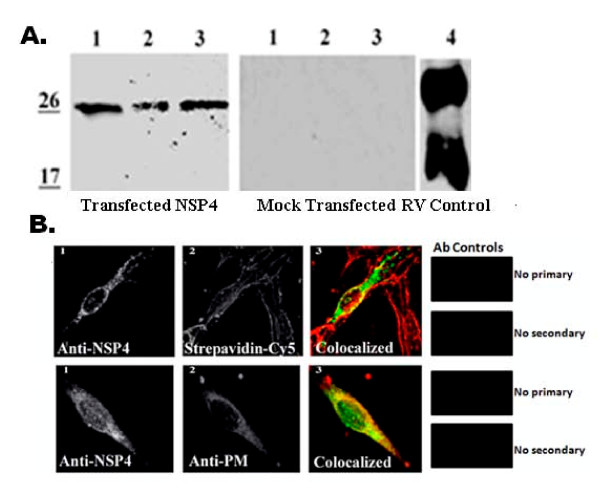
**Transfected NSP4 travels to the cell surface in the absence of other viral proteins**. **A**. BHK-21 cells were transfected with pcDNA3.2 NSP4_1-175 _and surface biotinylated at 20 h post transfection at 4°C. Cells were lysed and surface proteins were precipitated with streptavidin agarose (left panel). Mock transfected cells were treated identically and served as controls (middle panel). Lanes 1 are the cell lysates from transfected or un-transfected cells and lanes 2 are the surface biotinylated, streptavidin pull downs of the transfected (left) or mock-transfected (center) cells. Lanes 3 show the transfected or mock-transfected lysates following biotinylation and streptavidin pull-down. RV-infected cell lysate (right panel) is shown as a NSP4 control. Note that substrate with femtogram sensitivity was used to detect the expression of transfected NSP4. **B**. BHK-21 cells were grown on glass cover slips, transfected with pcDNA3.2 NSP4_1-175_, surface biotinylated in the cold, and probed with affinity-purified anti-NSP4_150-175 _and anti-rabbit IgG-CY2 (all panels) and either streptavidin-CY5 (top panels) or mouse anti- Na^+^/K^+^-ATPase and anti-mouse IgA-Texas Red (bottom panels,). Cells were visualized with a Stallion Digital Workstation. The colocalized images with surface molecules pseudo-colored red and NSP4 pixels pseudo-colored green are shown in panels 3. Yellow pixels indicate the areas of pixel overlap. On the far right is shown the antibody controls.

It was important to determine the extent RV replication or RV-encoded proteins contributed to NSP4 intracellular transport to, exposure on, and translocation across the PM. Due to low transfection efficiencies in the MDCK and HT29.F8 cultures, BHK-21 cells were utilized for these experiments. The BHK-21 cells were transfected with pcDNA3.2 NSP4_1-175 _and examined at 20 h post transfection by surface biotinylation followed by streptavidin precipitation and Western blot (Figure 8A) or fluorescent imaging (Figure 8B). Western blot analyses of the NSP4-transfected BHK-21 lysates of the mock biotinylated (lane 1) or post biotinylated/streptavidin pull-downs (lane 3) revealed a single, 28 kD band. The different glycosylated forms of NSP4 were not observed, similar to the infected lysates in the polarity experiment (above). In the streptavidin pull-downs of the transfected BHK-21 cells, the same FL, 28 kD NSP4 band was observed (Figure 8A, lane 2) indicating FL NSP4 was present on the exofacial membrane when expressed in the absence of other viral proteins. However, the quantity of NSP4 was considerably less than that seen in the infected cells. To sufficiently visualize the bands required the use of substrate with femtogram sensitivity. Whether the reduced quantity of NSP4 at the cell surface was due to the lack of virus or viral proteins or reduced expression of NSP4 will require additional study. Mock transfected cells (middle panel) and RV-infected lysates (right panel) are shown as controls.

Colocalizations of NSP4 and two markers, surface-bound biotin (with CY5-streptavidin) and mouse anti-Na^+^K^+^ATPase (PM marker, with Texas Red-conjugated anti-mouse IgG) in NSP4-transfected BHK-21 cells were completed to support the biotinylation/streptavidin pull-down data. The transfected BHK-21 cells were surface biotinylated at 20 hpi and probed with CY2-conjugated rabbit NSP4_150-175 _IgG to detect NSP4 and with CY5-streptavidin and Texas Red-conjugated anti-mouse IgG to detect the exofacial PM proteins (Figure 8B). Filter sets that were suitable for CY2, Texas Red and CY5 with a Stallion Digital Imaging Workstation were employed. The CY2-NSP4 signal was pseudo-colored green (panels 1 and 3) while the exofacial membrane indicator, the CY5-streptavidin (top panels 2 and 3) and the PM indicator, Texas Red-anti-mouse IgG (bottom panels 2 and 3) were pseudo-colored red. Evaluation of the number of cells with NSP4 fluorescence vs. the number lacking the NSP4 signal was utilized to estimate a transfection efficiency of 10-12%. Positive colocalizations of NSP4 with streptavidin and anti-Na^+^K^+^ATPase were observed as indicated by the presence of yellow pixels (panels 3). These data denote NSP4 travelled to and traversed the PM in the absence of virus or viral proteins, albeit at reduced levels.

### Soluble NSP4 released into culture media bound the exofacial surface of uninfected cells

**Figure 9:**
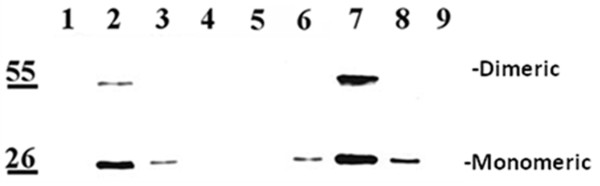
**Western Blot of surface biotinylated, uninfected MDCK and HT29.f8 cells in which media containing soluble NSP4 was exogenously added**. RV-free, NSP4-containing media exogenously was added to MDCK or HT29.F8 naïve cells and incubated for 90 min at 4°C. The treated cells were washed, surface biotinylated, lysed and the surface molecules precipitated with streptavidin agarose. The surface precipitates and lysates were separated by SDS-PAGE and analyzed by Western blot using anti-NSP4_150-175_. Dimeric and monomeric NSP4 are indicated. Lanes are as follows: 1 = MDCK lysates following the streptavidin pull-down. 2 = MDCK streptavidin pull-down (exofacial PM). 3 = MDCK cell lysates from the no biotin control. 4 = MDCK streptavidin pull-down from the no biotin control. 5 = Uninfected MDCK cell lysates. 6 = HT29.F8 lysates following the streptavidin pull-down. 7 = HT29.F8 streptavidin pull-down (exofacial PM). 8 = HT29.F8 cell lysates from the no biotin control. 9 = HT29.F8 streptavidin pull-down from the no biotin control

We hypothesized that the released NSP4 would associate with surface molecules on naïve cells, similar to that reported by Bugarcic and Taylor (2006) [51] and akin to the addition of purified NSP4 or peptide to the exofacial surface of cell monolayers or mouse intestinal mucosal [10]. Therefore the clarified, NSP4-containing media was exogenously added to naïve cells and incubated for 90 min at 4°C. The treated cells were washed, surface biotinylated, lysed and precipitated with streptavidin agarose or directly analyzed by Western blot prior to streptavidin addition (Figure 9). NSP4 was absent from the MDCK lysates following the precipitation of the surface molecules with streptavidin-agarose (lane 1), indicating that most of the added NSP4 was in the precipitate (lane 2). The streptavidin precipitate consistently showed two bands at 28 and 56 kD on the exofacial surface of both the MDCK (lane 2) and the HT29.F8 cells (lane 7) that corresponded to the FL, 28 kD, monomeric and dimeric NSP4. When the MDCK and HT29.F8 lysates were examined prior to precipitation, a single band at 28 kD was observed (lanes 3 and 8, respectively) signifying that NSP4 had bound the exofacial surface of the naïve cells in agreement with the presence of NSP4 in the streptavidin precipitates.

In the HT29.F8 cells, once the biotinylated surface proteins were removed from the lysates with streptavidin-agarose beads, the lysates were evaluated by Western blot to verify a lack of intracellular NSP4. Unexpectedly, a faint NSP4 band repeatedly was seen in the lysates after removal of the biotinylated proteins (lane 6). To ensure all of the biotinylated NSP4 was precipitated, we repeated the streptavidin pull downs 3 times prior to re-evaluating the lysate and acquired the same results. These data suggest a small amount of NSP4 was not available to the biotin reagent or a small amount of NSP4 entered the intestinal cells post exogenous addition.

This difference in PM permeability or surface availability to biotin of the exogenously added NSP4 in the HT29.F8 and MDCK cells requires additional study. Taken together, these experiments revealed the presence of biotinylated monomeric and dimeric NSP4 on the surface of intact, uninfected cells when treated with media from RV-infected cells, indicating the released NSP4 can bind surface molecules of neighboring cells to elicit its enterotoxic effects.

### Uninfected neighboring cells are bound by NSP4 at 3 and 7 hpi in culture

**Figure 10:**
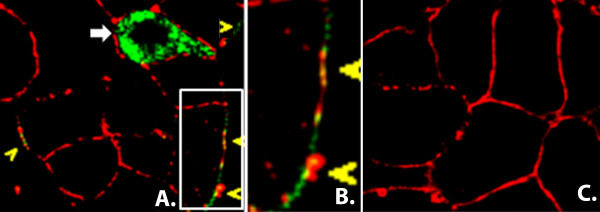
**Confocal analyses of RV-infected HT29.F8 cells at 7 hpi**. **Panel A**: Live HT29.F8 cells were surface biotinylated prior to fixation and permeabilization. Once the cells. were fixed and permeabilized, streptavidin-CY2 was utilized to label the biotinylated proteins on the exofacial PM (pseudo-colored red) and CY5-linked F(ab)_2 _prepared from anti-NSP4_150-175 _was added to label NSP4 (pseudo-colored green). The white, block arrow indicates an infected cell stained with anti-NSP4_150-175_-CY5-linked F(ab)_2_. The yellow arrows highlight the PM of the uninfected cells in which CY5-linked NSP4_150-175 _F(ab)_2 _is bound (green). **Panel B **is an enlargement of the white box in panel A and **Panel C **is the uninfected cell control that was labeled the same as the infected cells (panel A).

To examine the release and rebinding of NSP4 *in culture*, RV-infected (MOI = 2) and uninfected (control) HT29.F8 cells were surface biotinylated prior to fixation and permeabilization at 3 and 7 hpi. The infected and uninfected cells then were probed with CY2-streptavidin (pseudo-colored red) to detect all proteins on the exofacial PM and CY5-linked NSP4_150-175 _F(ab)_2 _(pseudo-colored green) to localize NSP4. A section of the slide was selected such that a single infected cell was apparent (NSP4 appears green) next to uninfected cells that only showed the red-stained PM (Figure 10A). The single infected cell (white arrow) showed several intracellular pools of NSP4 (green) with only a single point of co-localization (yellow) with the PM. The yellow arrows indicate areas of the exofacial PM where it appears that NSP4 had been secreted from an infected cell or cells and then rebound (green pixels) to the cell surface of uninfected, neighboring cells. The area in the white box (panel A) is enlarged and shown in panel B. There was no indication of NSP4 in mock-infected cells (panel C). At 3 hpi, no colocalization of NSP4 and PM was observed (not shown) supporting NSP4 was not released from infected cells at this early time point under the stated conditions. Further, these data support the presence of NSP4 on the surface of uninfected cells near an infected cell at 7 hpi, and indicate NSP4 was released from the infected cells to bind the exofacial PM of neighboring, uninfected cells. Note that NSP4 (pseudo-colored green) was apparent on the PM in sections if the membrane that lacked staining with the streptavidin (Panels A and B) indicating a lack or reduction of biotinylation in those areas.

## Discussion

Several reports indicate that the NSP4 enterotoxin is secreted from infected cells, yet each of these studies show a significant modification of the released glycoprotein. Bugarcic and Taylor (2006) detect a partially EndoH-resistant form of NSP4 with an approximate *Mr *of 32 kD in the media of intestinal-derived Caco-2 cultures infected with the bovine UK RV at an MOI of 10. In this study, the larger form of the glycoprotein is not apparent in infected lysates, but is the only size of NSP4 detected in media between 16 and 24 hpi [51]. In contrast, Zhang et al. (2000) show a 7 kD cleavage fragment (aa 112-175) of NSP4 is released from kidney-derived MA104 and intestine-derived parental HT.29 cultures when infected with the simian SA11 RV at a MOI of 20 [23]. In this study, FL NSP4 is detectable in lysates at 2.5 hpi and the 7 kD NSP4 fragment can be detected in media at 4 hpi [23]. Both of these studies conclude NSP4 secretion from the cell is dependent on secondary processing or modifications of the mature 28 kD glycoprotein. Our earlier data reveal FL, EndoH sensitive, 28 kD NSP4 is present in caveolae when isolated from a PM-enriched fraction devoid of detectable ER and fluid phase PM markers [48,73]. These differences likely are due to variation in the viral strain, MOI, times post infection when the cells were examined, the use of different cell lines, and most likely, a combination of these variables.

We report the exofacial exposure of a pool of NSP4 that was FL, EndoH-sensitive, and unmodified with respect to protease cleavage or the addition of unusual glycans in both intact MDCK and HT29.F8 cells. The same RV SA11.4F inoculum simultaneously was added to both cell types and analyses were completed at the same time, yet NSP4 transport kinetics and polarized sorting varied in the two cells. It is unknown how these cultured cells differ in terms of inherent transport mechanisms or interaction(s) with NSP4 or if the cells vary in the degree of soluble vs. vesicular trafficking, which would explain the kinetic differences [74].

An earlier report by Delmas, et al [75] documents a similar disparity in cells of a different origin when infected with RV. In this study VP4, the RV spike protein, is localized with rafts at the apical membrane, facilitates RV assembly and promotes non-lytic release of RV particles from the PM of the intestine-derived Caco-2 cells. In kidney MA104 cells, the RV particles are released by cell lysis despite RV and VP4 association with rafts in this cell line. Further analyses illustrate significant differences in gross lipid composition of the raft domains associated with VP4 in the two cell lines which may explain the different mechanisms of virus release [75]. This diversity in membrane rafts has been evaluated and confirmed in a recent review that sought to address key criticisms in the field, in particular the use of artificial criteria for defining raft domains such as detergent insolubility [76]. Rafts are best defined as dynamic, evolving cell membranes that begin as nanoscale assemblies of sterols, sphingolipids and proteins that fluctuate in composition and size [76-78]. These ordered nanoscale assemblies coalesce in response to signaling or trafficking events to form larger, functional raft domains via a variety of protein-lipid, lipid-lipid or protein-protein interactions [77,78]. A third, μm-sized raft state is observed only at equilibrium in isolated membranes [78]. The relationship(s) between these three states are unclear however the fluctuating behavior is dependent on the composition of the membrane, which are dependent on the cell type from which the PM is derived. Hence NSP4 association with rafts or other membrane domains can vary in different cells or the raft state. These events are further complicated by bilayer asymmetry, cortical actin nucleation of raft heterogeneity [79,80], and the lack of knowledge of raft assembly in the cytosolic leaflet, whose lipid content varies from the exofacial leaflet, which is also cell-dependent [81].

The data herein suggest that the NSP4 N-terminus was not modified or alternately processed, and therefore these alterations were not essential to NSP4 movement to the PM when tested under the described conditions. The secreted NSP4 recovered from culture media predominately was the same EndoH-sensitive, mature 28 kD form consistently and reproducibly seen at the cell surface of both SA11.4F RV-infected MDCK and HT29.F8 cultures. When media containing secreted NSP4 exogenously was added to uninfected cells, both the dimeric and monomeric forms were detected on the exofacial membrane of the naïve cells.

Similar kinetics of NSP4 and RV release implies that virus plays a role in NSP4 release. We did not anticipate the notable delay from when NSP4 reached the exofacial PM of the intestinal cells until it was detected in culture media. Possible explanations include (i) a lack of sensitivity such that NSP4 failed to be detected in the culture media until a sufficient quantity was accumulated; (ii) a threshold concentration of NSP4 at the PM was required before its release; (iii) NSP4 was secreted into the media but a significant portion of the secreted glycoprotein rebound the PM; or (iv) NSP4 was released with the virus. Additional studies are necessary to dissect the precise mechanism(s) of NSP4 secretion and the role(s) of virus in promoting the release of NSP4 from both cell types.

Another interesting finding was the exposure of the C-terminus on the exofacial surface of infected and NSP4-transfected cells. Detection of NSP4 on the cell surface prior to its release is reasonable given that the infected cells are intact and NSP4 would have to traverse the PM for its release. Both anti-NSP4_150-175 _and anti-NSP4_120-147 _bound the surface-exposed portion of NSP4. The anti-NSP4_120-147 _binding site has been mapped to aa 130-140 [27]. Together with the positive reactivity with anti-NSP4_150-175_, these data suggest that at least the C-terminal aa 130-175 are exposed on the exofacial membrane, prompting several questions. If NSP4 travels by a vesicular route from the ER, the budded vesicles likewise would have the C-terminus facing the cytosol. Upon vesicular fusion with the PM, the NSP4 N-terminus would appear on the exofacial surface while the C-terminus would remain cytosolic. This scenario is supported by the reactivity of the NSP4 C-terminus with cav-1, an cytofacial membrane-bound molecule [29,47]. The data herein suggest NSP4 'flips' its termini to expose the C-terminus to the exofacial membrane of the bilayer or NSP4 is a dual-topology homo-oligomer membrane protein [82]. If NSP4 travels via a soluble route, then the exposed termini could be somewhat random depending on the mechanism of translocation across the PM. One could envision the hydrophobic N-terminus interacting with the PM, again leaving the C-terminus cytosolic and requiring the protein to 'flip' to place the C-terminus facing outside of the cell. Additional studies are needed to dissect the precise mechanism(s) by which NSP4 traverses the PM.

In agreement with other studies [22-24,48,51], these data show NSP4 travels via a Golgi-bypassing, unconventional pathway. Nickel (2008) reports there are at least 4 distinct types of non-classical secretory export of proteins lacking an ER signal sequence but are still secreted to interact with receptors on adjacent target cells [83]. One or more of these mechanisms may apply to NSP4 and might begin to address the unique association(s) of NSP4 with the PM. The first type is typified by *Leishmania *surface protein HASPB in which the entire molecule traverses the PM while the protein remains membrane-anchored at the N-terminus [84,85]. This mechanism may require a flip-flop type of action [86]. The second type is the formation of PM exosomes or membrane blebbing that lyse in the extracellular fluid to release its content such as that used by galectins [87-89]. In this way, galectin is secreted prior to binding the cell surface [90]. This could account for the C-terminal exposure of NSP4 as the N-terminal membrane spanning domain would likely associate with the lipid bilayer. The other mechanisms include the use of specific transporters and entry into endolysosomes that fuse with the PM [83]. Finally, it has been proposed that partial unfolding could lead to an unfolded state called the molten globule state that is permissive to membrane translocation as seen with fibroblast growth factor 1 and IL-1 [91,92]. It remains to be determined if NSP4 trafficking and/or secretion is mediated by one or more of these reported mechanisms or by a unique process. We propose the process will vary dependent on the presence or absence of specific host-cell interacting molecules.

These data also have implications in RV pathogenesis. Exogenous introduction of purified NSP4 or the enterotoxic peptide (NSP4_114-135_) induces a phospholipase-C (PLC)-mediated, intracellular calcium [(Ca^2+^)i] mobilization resulting in chloride secretion and fluid accumulation in mouse pup intestines [10-12]. The enterotoxin could promote signaling events in the same infected cell or neighboring, uninfected cells via the exposure of the C-terminal enterotoxic domain and proximity to receptors. Alternatively, NSP4 may require release from the infected cell and subsequent binding to uninfected cells for the activation of early signaling events. Because RV escapes the intestine and can infect extraintestinal organs [43], examination of kidney and other cells becomes relevant.

Lastly this study showed variation in NSP4 expression and trafficking in cells of different origin. NSP4 is present in several organs outside of the intestine and has been shown to be associated with RV-associated histological lesions in the lungs and liver [43]. Although much work is needed to decipher the role of NSP4 in alternate tissues, this study suggests NSP4 functions differently in different cells and may contribute to viral pathogenesis in singular ways dependent on the cell type.

## Conclusions

To our knowledge, this is the first report of the presence of the C-terminus of FL, EndoH sensitive, NSP4 on the exofacial surface of RV-infected cells between 2-3 hpi (HT29.F8 cells) and 4-7 hpi (MDCK cells). Although NSP4 appeared the same by Western blot and was reactive to the same NSP4 C-terminal, peptide-specific antibodies, the transport kinetics was distinct and the directional transport was dissimilar when infected with the same RV inoculum. Surface expression of NSP4 was shown by three techniques, surface biotinylation-streptavidin precipitation, antibody binding to intact cells, and FRET by acceptor photobleaching. Each technique disclosed similar results, C-terminal NSP4 was exposed at the exofacial PM. Results from the transfected cells suggest NSP4 is sufficient to traffic to the cell surface but does not preclude an enhancement of NSP4 transport or release in the presence of virus or other viral proteins. EndoH sensitivity of the surface-exposed and secreted NSP4 signify the FL toxin bypassed the Golgi en route to the PM, remained on the cell surface long enough to be detected, and subsequently was released intact into culture media. It is curious that only the fully glycosylated NSP4 was observed on the cell surface, whereas multiple forms were seen in cell lysates, suggesting that glycans may play a role in transport and release. Together these data indicate the transport properties of NSP4 are complex, variable, and are dependent on a number of factors. Further examination of the mechanism(s) utilized by NSP4 for translocation across the PM, its orientation within the PM bilayer, as well as associations within the PM (transmembrane vs. peripheral, monomer vs. multimer, and chaperone vs. lipid annulus complexes) would provide valuable insights and potentially disclose alternate targets for vaccine and antiviral therapies.

## List of abbreviations

(aa): Amino acids; (DLP): double layered particles; (GADPH): d-glyceraldehyde-3-phosphate dehydrogenase; (EndoH): endoglycosidase H; (ER): endoplasmic reticulum; (ERGIC): ER-Golgi intermediate compartment; (*f_b_*): fraction of donors that are "bound" to an acceptor; (F_DA_): fluorescence intensity of the donor in the presence of the acceptor; (*f_u_*): fraction of "unbound" donors; (F_D_): fluorescence of the donor in the absence of acceptor; (FFU): focus forming units; (FL): full length; (FRET): fluorescence or Förster energy transfer; (HRPO): horse radish peroxidase; (hpi): hours post infection; (IDV): integrated density value; (kD): kilodaltons; (MOI): multiplicity of infection; (NSP4): non-structural protein 4; (PBS): phosphate buffered saline; (PM): plasma membrane; (PDI): protein disulfide isomerase; (*q_m_*): measured relative quantum yield resulting from FRET but containing background donor signal; (*q_u_*): quantum yield of the "unbound" donors not specifically involved in FRET, normalized to 1; (*r*): mean interaction distance between donor and acceptor pairs; (*R_0_*): Förster distance for fluorescent probes; (RV): rotaviruses.

## Competing interests

The authors declare that they have no competing interests.

## Authors' contributions

TFG completed the kinetics study, FRET analyses, RV growth curves, and release of NSP4 experiments. SMS initially showed the expression of NSP4 on cell surfaces at 24 hpi (starting point), and completed the surface antibody binding. CVW completed the transfection study. AM and FS contributed to all imaging results, FRET calculations and the intellectual design of the experiments. DMM showed the differential transport of NSP4 in polarized cells. RDP contributed to construction of the figures and to critically evaluating the manuscript, offering important intellectual content. JMB made significant contributions to the conception and design of the experiments, wrote the manuscript, and was instrumental in the critical assessments of the data and its interpretation. All authors have read and approve the final manuscript.
